# Virus-Derived Domesticated Genes in Microglia and Resident Macrophages: Insights into Placenta-Driven Evolution in Mammals

**DOI:** 10.3390/ijms27146322

**Published:** 2026-07-16

**Authors:** Masahito Irie, Fumitoshi Ishino, Tomoko Kaneko-Ishino

**Affiliations:** 1 Department of Epigenetics, Medical Research Institute (MRI), Tokyo Medical and Dental University (TMDU), Tokyo 113-8510, Japan; irie@dna-gib.com; 2 Faculty of Nursing, Tokai University School of Medicine, Isehara 259-1193, Japan

**Keywords:** virus-derived domesticated genes, microglia resident macrophage, metavirus, retrovirus, innate immune, placenta-driven evolution, germlines, preimplantation stages, low-DNA-methylation environments

## Abstract

Eutherian mammals possess 11 metavirus-derived genes, collectively known as the sushi-ichi retrotransposon homologue (SIRH)/Retrotransposon Gag-like (RTL) genes. Several members of this group function in microglia, where *SIRH3/RTL6*, *SIRH8/RTL5*, and *SIRH10/RTL9* mediate innate immune responses against bacterial-, viral-, and fungal-derived pathogen-associated molecular patterns, respectively, whereas *SIRH11/RTL4* responds to noradrenaline and is thought to contribute to stress-responsive brain functions. In addition, the retroviral *Env*-derived gene *ERVPb1* has been implicated in yolk sac-derived resident macrophage lineages, suggesting that both *Gag*- and *Env*-derived domesticated genes contributed to the evolution of mammalian neuroimmune systems. Microglia are now recognized as central regulators of neural circuit formation, brain homeostasis, and neuroimmune function, and their dysfunction has been implicated in a wide range of neurological and psychiatric disorders. Here, we review current knowledge of virus-derived genes that have shaped the functional evolution of microglia. We also revisit the concept of “Placenta-driven evolution,” which proposes that the relatively hypomethylated developmental environment of extraembryonic tissues, including the placenta and yolk sac, facilitates the retention and functional co-option of virus-derived sequences, thereby accelerating eutherian evolution. Finally, by integrating recent advances in virus-derived genes and retroelement biology, we discuss how this evolutionary framework may extend beyond extraembryonic tissues to other hypomethylated developmental environments, including the germline and preimplantation embryos.

## 1. Introduction

The human genome contains approximately 8–10% long terminal repeat (LTR) retrotransposons and endogenous retroviruses (ERVs) [1,2]. ERVs are LTR-type retroelements, originating from ancestral retroviral infections that became fixed in the host genome and subsequently lost most of their transpositional activity. Importantly, some of these elements have been co-opted by the host as domesticated genes with physiological functions. The discovery of the retroviral *Env*-derived *syncytin* gene in 2000 [3,4], followed by the identification of Paternally expressed 10 (*PEG10*) and *PEG11*/Retrotransposon Gag-like 1 (*RTL1*), derived from the sushi-ichi LTR retrotransposon in 2001 [5,6], gave rise to the concept that virus-derived genes may have contributed to the evolution of the mammalian placenta [7,8,9,10,11,12,13]. Together, these discoveries established that virus-derived genes are important contributors to mammalian evolution.

The sushi-ichi retrotransposon belongs to the Ty3/gypsy group of LTR retrotransposons, also referred to as the metavirus lineage [14,15]. Sushi-ichi-like elements are widely distributed from fish to birds; however, active sushi-ichi-like retrotransposons have not been identified in mammalian genomes. This observation strongly suggests that the sushi-ichi retrotransposon homologues (SIRH)/RTL genes, including *PEG10* and *PEG11/RTL1*, derived from ancient sushi-ichi-like metavirus elements that were domesticated during mammalian evolution. Phylogenetic analyses further suggest that *PEG10*, *PEG11/RTL1*, and the remaining SIRH/RTL genes were acquired through at least three independent domestication events [15]. Therefore, in this review, we refer to the SIRH/RTL genes collectively as “metavirus-derived genes.”

*PEG10* is conserved in both marsupials and eutherians [16], whereas the other SIRH/RTL genes are specific to eutherian mammals [11,17,18]. Recent studies have demonstrated that *PEG10* and *PEG11/RTL1*, best known for their placental functions, also function in the nervous system [19,20,21,22,23]. In contrast, four other SIRH/RTL genes—*SIRH3/RTL6*, *SIRH8/RTL5*, *SIRH10/RTL9*, and *SIRH11/RTL4*—have been shown to function predominantly in microglia within the brain. Analyses using knock-in (KI) mice expressing fluorescently tagged endogenous proteins revealed that *SIRH3/RTL6*, *SIRH8/RTL5*, and *SIRH10/RTL9* participate in innate immune responses against bacterial lipopolysaccharide (LPS), viral double-stranded RNA (dsRNA), and fungal-derived zymosan, respectively [24,25,26,27]. In addition, *SIRH11/RTL4* has been implicated in the regulation of microglial responses to the neurotransmitter noradrenaline (NA) and may contribute to stress-responsive neural regulation [28,29].

Microglia are now recognized not only as innate immune cells of the central nervous system but also as key regulators of synaptic pruning, elimination of unnecessary neurons, neural circuit formation, and activity-dependent modulation of the brain environment [30,31,32,33]. Dysregulation of microglial functions has also been increasingly implicated in neurodegenerative and psychiatric disorders [30,34,35]. Intriguingly, many metavirus-derived genes appear to function in both the placenta and the brain, two hallmark innovations of mammalian evolution [11,13,36]. These findings suggest that virus-derived genes have broadly contributed to the evolution of mammalian reproductive, neural, and immune systems [36]. In this review, we summarize the functions of virus-derived domesticated genes, including the eutherian-specific metavirus-derived SIRH/RTL genes, in microglia and resident macrophage lineages, and discuss their evolutionary significance. We further revisit the placenta-driven evolution hypothesis [36] by integrating recent findings from the germline and preimplantation embryo, thereby extending this framework to encompass a broader range of hypomethylated developmental environments involved in the retention and functional domestication of virus-derived sequences during eutherian evolution.

## 2. Virus-Derived Domesticated Genes Functioning in Yolk Sac-Derived Resident Macrophage Lineages

### 2.1. SIRH3/RTL6 and SIRH8/RTL5: Metaviral Gag-Derived Innate Immune Genes in Eutherians

**Figure 1:**
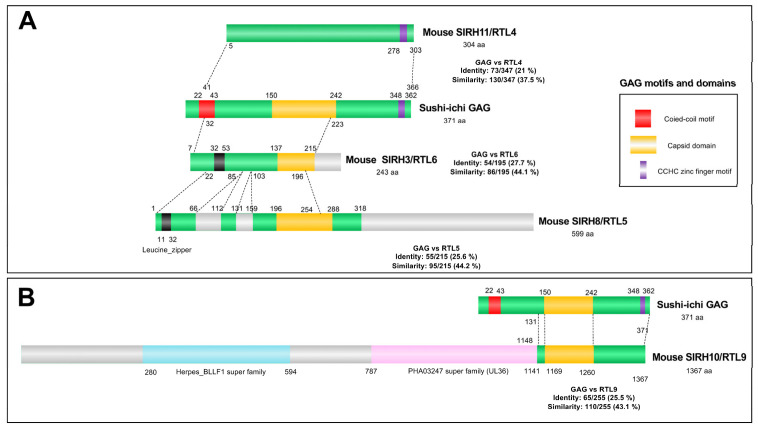
Homology of four SIRH/RTL proteins with sushi-ichi GAG: (**A**) Alignment of the sushi-ichi GAG, mouse SIRH3/RTL6, SIRH8/RTL5, and SIRH11/RTL4 proteins. SIRH3/RTL6 and SIRH8/RTL5 lack the coiled-coil motif and CCHC zinc finger motifs but possess a new leucine zipper motif at their N-termini (shown in black boxes). The gray boxes indicate an absence of any homology with the GAG protein. SIRH11/RTL4 lacks the coiled-coil motif and the capsid domain but retains the CCHC zinc finger motif. (**B**) Alignment of the sushi-ichi GAG and mouse SIRH10/RTL9 protein. SIRH10/RTL9 has a GAG-like structure only at its C-terminus and contains two retroviral structures: the Herpes BLLF1 superfamily motif (light blue) and the PHA03247 (UL36) superfamily motif (pink).

*SIRH3/RTL6* is an exceptionally well-conserved gene among eutherian mammals [24,36]. The dN/dS ratio of *SIRH/RTL6* (<0.05) is far lower than the reported average value for typical housekeeping genes (0.093) [37], suggesting that *SIRH3/RTL6* has been maintained under strong purifying selection throughout eutherian evolution. *SIRH8/RTL5* is the phylogenetically closest paralog of *SIRH3/RTL6* and appears to encode a substantially larger protein containing additional inserted regions relative to *SIRH3/RTL6* (Figure 1A). The dN/dS values of *SIRH8/RTL5* (0.2–0.5) also indicate negative selection. However, this gene has not been detected in several eutherian species, possibly due in part to incomplete genome assemblies [24]. Using recent updated genome assemblies, we confirmed pseudogenization of *SIRH8/RTL5* in certain groups of eutherians, a very limited number of Laurasiatherian species, several Afrotherian species, and most Xenarthran species (see Section 3 and Supplementary Data S1).

**Figure 2:**
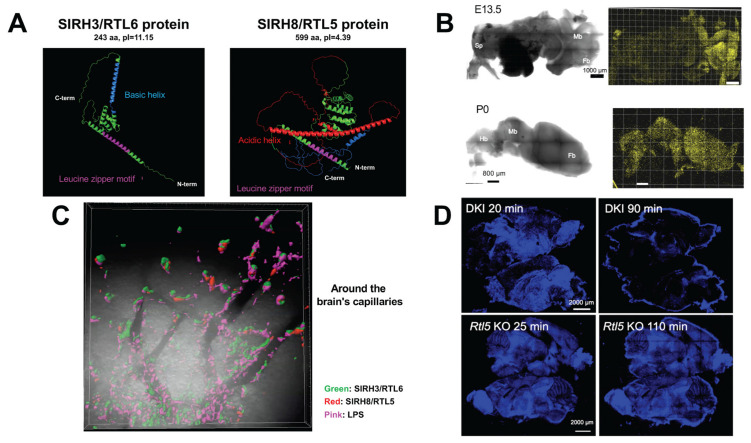
SIRH3/RTL6 and SIRH8/RTL5: (**A**) Three-dimensional structure predictions of the SIRH3/RTL6 and SIRH8/RTL5 proteins. Pink: the leucin-zipper motif, red: acidic regions, blue: basic regions. (**B**) Expression of SIRH3/RTL6 in the central nervous system at E13.5 and P0. Yellow: Venus signals. Fb: frontal brain; Hb: hindbrain; Mb: midbrain; Sp: spinal cord. (**C**) The SIRH3/RTL6-LPS complex formed near the brain’s capillaries. SIRH3/RTL6 aggregates to prevent LPS from accumulating in brain capillaries. When SIRH8/RTL5 joins this complex, larger complexes form and move away from the capillaries. (**D**) SIRH8/RTL5 is involved in dsRNA degradation. In *Sirh3/Rtl6* and *Sirh8/Rtl5* double KI (DKI) mice that have functional SIRH3/RTL6 and SIRH8/RTL5, almost all of the dsRNA administered into the brain is degraded after 90 min. By contrast, the signal remains unchanged in *Rtl5* KO mice, even after 110 min. These figures are taken from Reference [24].

Both *SIRH3/RTL6* and *SIRH8/RTL5* encode proteins homologous to retroviral GAG proteins (Figure 1A). The 243-amino-acid SIRH3/RTL6 protein contains a C-terminal helix enriched in basic amino acids and is highly basic overall, with a predicted isoelectric point (pI) of 11.15 (Figure 2A, left). In contrast, the 599-amino-acid SIRH8/RTL5 protein is strongly acidic overall (predicted pI 4.39) due to its strongly acidic helices, while also retaining a similarly basic C-terminal region (Figure 2A, right). In addition, both proteins possess canonical leucine zipper structures at their N-termini, suggesting an intrinsic capacity for dimerization or higher-order multimerization (Figure 2A) [24].

To determine the localization of SIRH3/RTL6 in vivo, KI mice expressing a Venus-tagged SIRH3/RTL6 protein from the endogenous *Sirh3/Rtl6* locus were generated [24]. Fluorescent puncta were broadly detected throughout the central nervous system beginning in middle embryogenesis, with the strongest signals observed during the neonatal period (Figure 2B) [24]. Similar punctate signals were also observed in brain microglia and in primary cultured microglia, suggesting that SIRH3/RTL6 is expressed in microglia and distributed within the extracellular space of the brain. Notably, following intracerebral administration of lipopolysaccharide (LPS), the diffusely distributed SIRH3/RTL6 signals rapidly accumulated at the injection site and formed large granular structures. Confocal laser microscopy demonstrated that LPS co-localized within these SIRH3/RTL6-positive assemblies (Figure 2C). These observations suggest that SIRH3/RTL6 is broadly distributed throughout the brain and may provide an immediate extracellular defense mechanism by locally sequestering invading pathogen-associated molecular patterns (PAMPs) from bacteria [24,26,27].

Similarly, analyses using KI mice expressing a SIRH8/RTL5-mCherry fusion protein from the endogenous *Sirh8/Rtl5* locus demonstrated that SIRH8/RTL5 responds to viral-like PAMPs, including double-stranded RNA (dsRNA) homologs and unmethylated CpG DNA [24,26,27]. Furthermore, analyses using double KI (DKI) mice expressing both SIRH3/RTL6-Venus and SIRH8/RTL5-mCherry revealed that SIRH8/RTL5 was also incorporated into the same LPS-induced granular assemblies (Figure 2C). Functional analyses further demonstrated that *Sirh3/Rtl6* knockout (KO) mice exhibited impaired clearance of LPS from the brain. The LPS signals were rapidly cleared (within 20 min) from the WT and KI mice, whereas they persisted for at least approximately 2 h in KO mice [24]. Similarly, *Sirh8/Rtl5* KO mice showed impaired clearance of dsRNA (Figure 2D) [24]. These findings suggest that SIRH3/RTL6 and SIRH8/RTL5 contribute to the elimination of bacterial- and viral-derived PAMPs from the brain [24,26,27].

The ancestral GAG proteins from which SIRH3/RTL6 and SIRH8/RTL5 were derived originally functioned as self-assembling viral capsid proteins. The highly basic C-terminal helical region acquired by SIRH3/RTL6 may have evolved to enhance interactions with negatively charged molecules such as LPS (Figure 2A). In addition, the N-terminal leucine zipper structures likely facilitate dimerization and formation of higher-order assemblies (Figure 2A,C) [24]. Although SIRH8/RTL5 contains extensive acidic regions internally, it also retains a highly basic C-terminal region similar to that of SIRH3/RTL6, suggesting that it may interact with PAMPs possessing diverse electrostatic properties. Furthermore, SIRH8/RTL5 may cooperate with SIRH3/RTL6 in the formation of larger extracellular PAMP-sequestering assemblies within the brain (Figure 2B,C) [24].

### 2.2. SIRH10/RTL9: Metaviral Gag-Derived Innate Immune Gene in Eutherians

*SIRH10/RTL9* is also conserved across all examined eutherian mammals, with dN/dS values ranging from 0.4 to 0.77 [25]. Structurally, however, *SIRH10/RTL9* differs markedly from the other SIRH/RTL genes. More than two-thirds of the protein consists of regions homologous to two distinct herpesvirus-like sequences, whereas the Gag-derived region is restricted to the C-terminal portion (Figure 1B).

**Figure 3:**
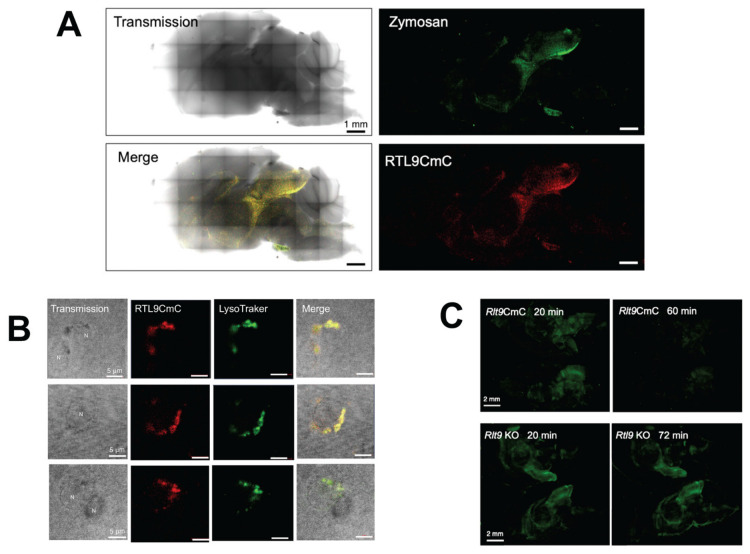
SIRH10/RTL9: (**A**) The RTL9 protein with mCherry (RTL9CmC) is found at the zymosan injection site. The right figure shows that red represents the mCherry signal and green represents the zymosan autofluorescence signal (artificial color). (**B**) RTL9CmC co-localizes with zymosan in the lysosomes of microglia. N: nucleus. Red: mCherry signal. Green: LysoTracker signal. (**C**) SIRH10/RTL9 is involved in zymosan degradation. After 60 min, nearly all of the zymosan introduced into the brains of WT (*Rtl9*CmC) mice is degraded. In contrast, the signal remains unchanged *in Rtl9* KO mice, even after 72 min. These figures are taken from Reference [25].

Analyses using KI mice expressing a SIRH10/RTL9-mCherry fusion protein from the endogenous *Sirh10/Rtl9* locus demonstrated that SIRH10/RTL9 is also expressed in microglia [25]. Following intracerebral administration of zymosan, a fungal-derived PAMP [26,27], SIRH10/RTL9-mCherry signals co-localized with zymosan signals (Figure 3A). In contrast to SIRH3/RTL6 and SIRH8/RTL5, which are distributed extracellularly, SIRH10/RTL9 was predominantly localized within microglial lysosomes (Figure 3B). In *Sirh10/Rtl9* KO mice, zymosan signals persisted in the brain after administration, suggesting that SIRH10/RTL9 contributes to the clearance of fungal-derived PAMPs in the brain (Figure 3C).

Zymosan is known to bind Dectin-1 and Mac-1 on the microglial surface, leading to phagocytic uptake and subsequent fusion with lysosomes to form phagolysosomes, where degradation occurs [38,39,40]. Lysosomes are intracellular organelles responsible not only for the degradation of PAMPs but also for the breakdown of insoluble intracellular materials, and their internal environment is maintained under highly acidic conditions [41,42]. The characteristic mosaic-like domain organization of SIRH10/RTL9 may have evolved to enable stable function within these specialized lysosomal conditions.

### 2.3. SIRH11/RTL4: Metaviral Gag-Derived Gene Associated with Noradrenergic Stress Signaling in the Brain

**Figure 4:**
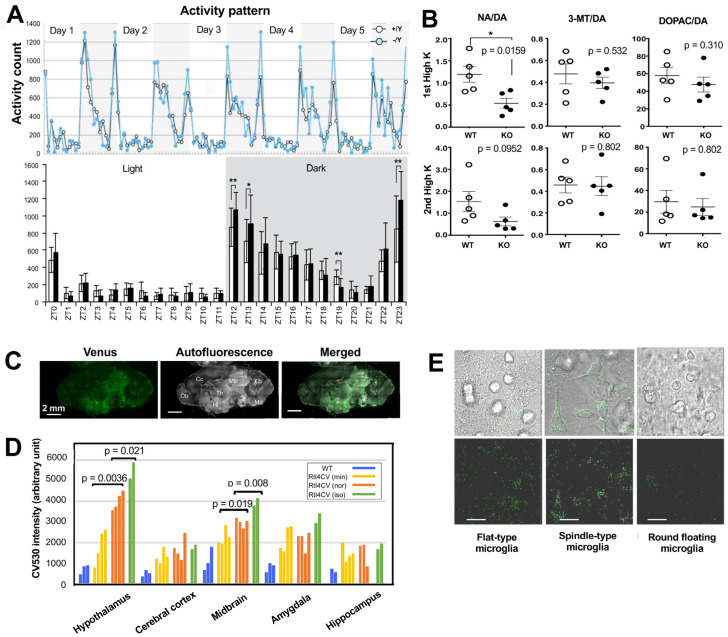
SIRH11/RTL4: (**A**) *Sirh11/Rtl4* KO mice show reduced adaptability to new environments. Although activity increases during light–dark cycle transitions, WT mice exhibit reduced activity by the second day, indicating adaptation. In contrast, KO mice exhibit the same reactions observed on the first day throughout the five-day period in a new environment. Statistically significant difference below 0.05 (*) and below 0.01 (**). (**B**) NA recovery in the frontal lobe of *Sirh11/Rtl4* KO mice is delayed. There are no changes in 3-MT or DOPAC, which are dopamine (DA) metabolites other than NA. (**C**) SIRH11/RTL4 protein expression in the brain. Cb: cerebellum, Cc: cerebral cortex, Ht: hypothalamus, Mb: midbrain, Md: medulla oblongata, Ob: olfactory bulb, Th: thalamus. (**D**) The intensity of SIRH11/RTL4 signaling increases under stress. In the figure below, blue indicates the signal intensity (autofluorescence) of the wild type (WT). Yellow represents the stress-free state in the animal housing room, orange represents the state after transport from the housing room to the laboratory, and green represents the state after the administration of isoproterenol, a NA agonist. (min): minimal stress condition, (nor): normal condition, (iso): isoproterenol administration. (**E**) SIRH11/RTL4 protein is expressed in microglia. In primary microglial cultures, Venus (green) signaling is observed in three types of microglial cells: the flat type within the feeder cell (astrocyte) layer, the spindle-shaped type on the surface of that layer, and the round type floating in the upper layer. Scale bar: 2 mm (**A**,**B**) are adapted from Reference [28], and (**C**,**D**) are adapted from Reference [29].

*Sirh11/Rtl4* KO mice exhibit behavioral abnormalities, including increased impulsivity, impaired adaptation to novel environments, and deficits in short-term spatial memory, in addition to the delayed recovery of NA, suggesting an important role for *SIRH11/RTL4* in brain function (Figure 4A,B) [28]. In humans, *SIRH11/RTL4* (also known as *ZCCHC16*) has also been reported as a rare autism spectrum disorder (ASD)-associated gene, further implicating it in neuropsychiatric functions [43].

Analyses using KI mice expressing a SIRH11/RTL4-Venus fusion protein from the endogenous *Sirh11/Rtl4* locus revealed that SIRH11/RTL4 is predominantly expressed in the hypothalamus, midbrain, and amygdala (Figure 4C) [29]. Interestingly, the SIRH11/RTL4-Venus signal increased markedly in response to environmental stress and isoproterenol administration, a β-adrenergic receptor agonist that mimics noradrenergic signaling (Figure 4D). The signal was also highly dependent on the state of arousal; within five minutes of isoflurane anesthesia, it rapidly declined to basal levels. In contrast, the administration of the NA reuptake inhibitor milnacipran suppressed the anesthesia-induced reduction. Together, these results suggest that SIRH11/RTL4 reflects NA levels in the brain [29].

Unlike the other three microglia-associated SIRH/RTL genes, which participate in the elimination of PAMPs, SIRH11/RTL4 does not appear to function directly in pathogen defense. Instead, its expression in microglia (Figure 4E) suggests a role in regulating neuroimmune homeostasis. Because NA regulates microglial activation, while excessive NA signaling can be neurotoxic [44,45], rapid restoration of extracellular NA homeostasis is therefore likely to be important. Under normal conditions, extracellular NA is promptly removed through reuptake mechanisms. However, delayed recovery of the NA level in the prefrontal cortex has been observed in *Sirh11/Rtl4* KO mice [28], suggesting that SIRH11/RTL4 may contribute to the regulation of NA-associated neuroimmune responses through direct interaction with NA and/or modulation of its reuptake process. Taken together, these findings suggest that SIRH11/RTL4 functions not only in maintaining NA homeostasis but also in fine-tuning neuroimmune responsiveness through regulation of NA signaling. This role is conceptually analogous to those of *SIRH3/RTL6*, *SIRH8/RTL5*, and *SIRH10/RTL9*, which maintain brain homeostasis by promoting the clearance of PAMPs. In addition, SIRH11/RTL4 may contribute to the clearance of DAMPs, including nucleic acids released from apoptotic neurons through its conserved C-terminal CCHC RNA-binding motif [29]. Collectively, these findings identify SIRH11/RTL4 as the first reported virus-derived, microglia-associated gene implicated in ASD-related phenotypes and further expand the repertoire of metavirus-derived genes functioning in microglia beyond innate immunity to include noradrenergic neuroimmune regulation.

### 2.4. ERVPb1: Retroviral Env-Derived Gene in Simiiform Primates

**Figure 5:**
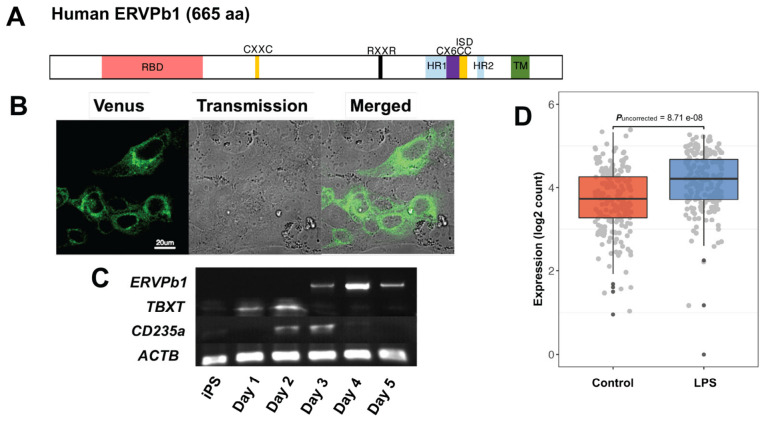
ERVPb1: (**A**) The human ERVPb1 protein (665 aa) shows 70.3% sequence identity with the HERVIP10B3 ENV protein (670 aa) and retains the basic motifs of the ENV protein: The motifs include CXXC and CX5CC (disulfide-bond isomerase), HR1 and HR2 (heptad repeats), ISD (immunosuppressive domain), and RBD (receptor-binding domain). TM is the transmembrane domain. (**B**) ERVPb1 is expressed in macrophage-like cells. Green: Venus signal. Venus is fused to the 48th amino acid from the N-terminus of ERVPb1. (**C**) *ERVPb1* is expressed following *TBXT* (a mesoderm marker) and *CD235a* (a hemangioblast marker) when embryoid bodies generated from iPS cells are placed in differentiation medium. (**D**) Transcript levels of the human *ERVPb1* region increase when monocytes are treated with LPS [46,50]. These figures are cited from Reference [46].

*ERVPb1* is a retroviral *Env*-derived domesticated gene conserved among simiiform primates, including humans, and retains an almost full-length ENV ORF (Figure 5A) [46]. However, unlike *syncytin*-related genes, no clear placental expression has been detected, and fusogenic activity has not been reported [47,48].

To investigate its expression profile, human iPS cells carrying a Venus fluorescent protein fused immediately downstream of the N-terminus of the endogenous *ERVPb1* locus were generated and differentiated through an embryoid body (EB)-based system [46]. Fluorescence analyses revealed ERVPb1 expression in cells exhibiting macrophage-like morphology (Figure 5B). Furthermore, when these EBs were cultured under macrophage differentiation conditions, ERVPb1-positive cells emerged at an unusually early stage, within 3–5 days after induction (Figure 5C). Since conventional human iPS cell-derived macrophage differentiation systems generally require more than 2–3 weeks to generate mature macrophages [49], this early appearance suggests that ERVPb1-expressing cells may possess characteristics resembling primitive yolk sac-derived macrophage lineages [46].

In addition, genome-wide association studies (GWAS) in human populations have identified associations between the *ERVPb1* locus and immune-related traits, including responsiveness to LPS in monocytes (Figure 5D) [46,50]. These findings raise the possibility that *ERVPb1* participates in innate immune responses in resident macrophage lineages in humans.

To date, retroviral *Env*-derived domesticated genes such as *syncytins* and *suppressyn* have been studied primarily for their roles in placental development and regulation of cell fusion [9,10,11,12,51]. *ERVPb1*, in contrast, represents an *Env*-derived gene that may function in resident macrophage lineages, similarly to the SIRH/RTL genes described above. Whether *ERVPb1* is also expressed in microglia remains unknown. Nevertheless, these findings suggest that a subset of retroviral *Env*-derived genes may have been co-opted not only for placental evolution but also for the evolution and diversification of yolk sac-derived resident macrophage lineages.

## 3. Discussion

### 3.1. Functional Diversification of Virus-Derived Genes in Mammalian Microglia

Microglia are a specialized population of resident macrophages that, unlike bone marrow-derived macrophages, originate from primitive yolk sac-derived macrophage lineages during early embryogenesis and subsequently colonize the brain [52,53]. Although macrophages themselves are broadly conserved throughout the animal kingdom, the presence of embryonically derived resident macrophages within the central nervous system is widely conserved among vertebrates [54]. Microglia possess a shared core transcriptional program and play essential roles not only in innate immunity but also in neural circuit formation and maintenance of the brain environment through interactions with neurons and other glial cells [30,31,32,33,34,35,53,54,55]. These conserved features suggest that microglia contributed substantially to the emergence of highly organized neural circuits and activity-dependent brain functions during mammalian evolution [55].

**Figure 6:**
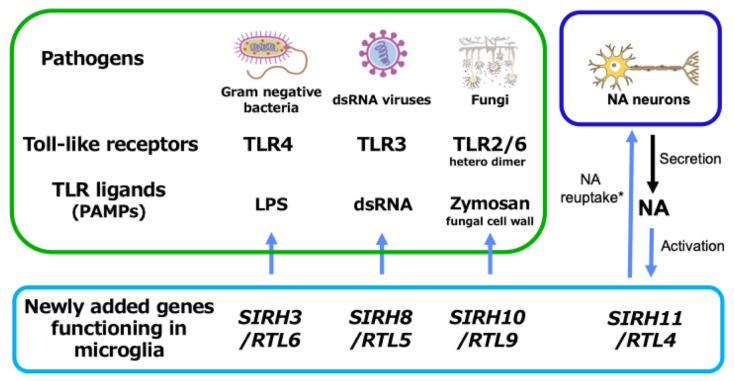
Proposed functions of metavirus-derived SIRH/RTL genes in microglia: *SIRH3/RTL6*, *SIRH8/RTL5*, and *SIRH10/RTL9* play important roles in innate immunity by promoting the clearance of pathogen-associated molecular patterns (PAMPs), whereas *SIRH11/RTL4* functions in NA-dependent neuroimmune regulation. Their evolutionary distribution among eutherian mammals further raises the possibility that *SIRH11/RTL4* acts as a regulator that fine-tunes the sensitivity of neuroimmune responses to environmental changes during eutherian evolution. * Predicted function.

For newborn mammals encountering the external environment for the first time at birth, exposure to bacteria, viruses, and fungi would have represented a major evolutionary threat. Thus, the PAMP clearance mechanisms mediated by *SIRH3/RTL6*, *SIRH8/RTL5*, and *SIRH10/RTL9* may have provided important adaptive advantages during eutherian evolution [24,36] (Figure 6). Likewise, the acquisition of *SIRH11/RTL4* may have protected the brain from excessive NA signaling. It may also have enhanced the ability of eutherian mammals to respond rapidly to environmental changes and to adapt to novel environments through modulation of NA-dependent microglial functions [28,29]. Collectively, these domesticated metavirus-derived genes are unique to eutherian mammals and likely contribute substantially to the emergence of the sophisticated neuroimmune systems characteristic of eutherian mammals [11,13,36] (Figure 6).

Notably, *SIRH3/RTL6* and *SIRH10/RTL9* are conserved among all eutherian species, whereas *SIRH8/RTL5* has been lost in several mammalian lineages, including most species within Xenarthrans, several species within Afrotherians, and a limited number of species within Laurasiatherians, particularly within Chiroptera and Eulipotyphla [24] (Supplementary Data S1). Interestingly, bats possess a unique immune system that enables them to harbor numerous viruses without developing overt disease. Comparative genomic analyses suggest that modifications of DNA damage-response pathways during the evolution of powered flight also reshaped bat immune systems, as these pathways play central roles in host defense and are frequent targets of viral interactions [56,57,58]. In contrast, Xenarthrans are characterized by unusually low body temperatures and basal metabolic rates, typically only 40–60% of those predicted for mammals of comparable body mass [59,60]. Because energetically costly immune responses, particularly inflammation, impose substantial metabolic demands, Xenarthrans may have evolved to rely more heavily on disease tolerance—rather than pathogen elimination—as a strategy for host defense [61,62]. Collectively, these observations indicate that lineage-specific ecological and physiological adaptations have influenced the evolutionary requirements for virus-derived immune genes.

Similarly, *SIRH11/RTL4*, a noradrenaline-responsive gene, has also undergone lineage-specific evolutionary modifications in several mammalian groups [28,63]. In Xenarthrans, *SIRH11/RTL4* has been reported to be pseudogenized in sloths and armadillos through the accumulation of mutations [28]. Our reanalysis of the latest genomic data further demonstrates that *SIRH11/RTL4* is also pseudogenized in anteaters, indicating that pseudogenization has occurred throughout all three extant orders in Xenarthrans (Supplementary Data S2). In Cetaceans and Ruminants, approximately the N-terminal half of the protein has been deleted, whereas in megabats and several other species, deletions affecting the C-terminal CCHC motif within the RNA-binding domain have been identified [63]. These findings raise the possibility that in Xenarthrans, the pseudogenization of *SIRH11/RTL4* may have reduced noradrenaline-dependent microglial activation, thereby minimizing energetically costly neuroinflammatory responses in association with their characteristically low metabolic rate and low activity. In contrast, in megabats, the loss of the C-terminal RNA-binding domain of SIRH11/RTL4 may represent functional remodeling that attenuates excessive neuroimmune activation from NA during repeated sympathetic stimulation associated with sustained flight, while preserving other aspects of RTL4 function. Together, these observations suggest that *SIRH11/RTL4* functions have undergone evolutionary diversification in accordance with species-specific physiological, ecological, and behavioral adaptations [63]. Accordingly, these observations also raise the possibility that, rather than simply mediating NA signaling, SIRH11/RTLA4 functions as a regulator that fine-tunes the sensitivity of neuroimmune responses to environmental changes, thereby facilitating adaptation to changing or novel environments. Accordingly, lineage-specific retention, structural remodeling, or loss of SIRH11/RTL4 may represent evolutionary optimization of neuroimmune responsiveness in response to distinct ecological, physiological, and behavioral demands [63]. Despite partial loss or structural divergence in several mammalian lineages, the strong conservation of SIRH11/RTL4 in humans and mice suggests that its neuroimmune functions have remained under purifying selection in many eutherian lineages [28,29].

Collectively, these findings support the view that virus-derived RTL genes function as evolutionarily flexible regulators rather than fixed antiviral effectors. Their retention, functional modification, or loss appears to have been shaped by lineage-specific ecological, physiological, and metabolic constraints, thereby optimizing immune and neuroimmune homeostasis according to the life-history strategies of individual mammalian lineages.

Further comparative studies will be necessary to clarify how these eutherian-specific metavirus-derived genes have contributed to the functional evolution of eutherian microglia in more detail. Unexpectedly, recent RNA-seq analyses of microglia derived from *Sirh3/Rtl6* KO mice reveal that the SIRH3/RTL6 protein is an essential factor in the TLR4 downstream signaling pathway in response to LPS [64,65,66,67,68]. Moreover, it appears to play an important role in maintaining microglial interactions with neurons and other glial cells while also regulating cellular proliferative machinery. These findings imply that metavirus-derived *SIRH3/RTL6* has broader function than previously recognized. They further suggest that SIRH3/RTL6 serves as a key regulator of the microglial state in innate immune systems of extant eutherian mammals rather than functioning solely as an LPS recognition molecule [24,64]. In addition, our ongoing RNA-seq analyses suggest altered microglia–neuron interactions in *Sirh11/Rtl4* KO microglia. These observations raise the possibility that *SIRH11/RTL4* also contributes to the regulation of microglial state, consistent with its proposed role in fine-tuning neuroimmune responsiveness to environmental changes. Future comparative transcriptomic and functional analyses of individual SIRH/RTL KO models will therefore be essential for understanding how these eutherian-specific virus-derived genes collectively shaped the evolutionary diversification of microglial functions.

The discovery that *ERVPb1*, an ENV-derived gene, is expressed in resident macrophages revealed that the functions of ENV-derived genes extend beyond their well-established role in placental cell fusion [46]. Furthermore, *ERVPb1* expression is induced by LPS stimulation in human primary monocytes [46,50], providing an intriguing parallel with the GAG-derived SIRH/RTL genes that function in microglia. Although *ERVPb1* and the SIRH/RTL genes originated from distinct viral proteins with fundamentally different molecular properties—ENV functioning as a membrane protein and GAG-derived proteins functioning predominantly in the cytoplasm or extracellular space—both appear to have been recruited into macrophage biology. Together, these findings suggest that virus-derived genes originating from distinct viral structural proteins have convergently evolved to regulate immune and neuroimmune functions in mammalian macrophage lineages. More broadly, these observations raise the possibility that independent classes of virus-derived genes have been repeatedly recruited into macrophage biology during mammalian evolution.

### 3.2. Hypomethylated Developmental Environments and Virus-Derived Gene Domestication

Importantly, virus-derived domesticated genes are found not only among genes functioning in the placenta [11,12,13], but also among genes functioning in yolk sac-derived resident macrophage lineages [24,25,29,46]. Based on this observation, we previously proposed the concept of “Placenta-driven evolution,” in which virus-derived genes that could function in hypomethylated extraembryonic tissues, such as placenta and yolk sac, were preferentially retained and co-opted during mammalian evolution [36]. It is particularly significant that eutherian-specific virus-derived genes are expressed in the placenta and the brain, two hallmark innovations of eutherian mammals. This observation raises the possibility that virus-derived genes contributed not only to placental evolution but also to the functional diversification of the mammalian brain [36].

According to this hypothesis, the acquisition of virus-derived genes that enabled placental evolution created opportunities for the further recruitment of virus-derived sequences, thereby accelerating eutherian evolution. Although named after the placenta, which serves as the most prominent symbol of this process, the hypothesis proposes that hypomethylated extraembryonic tissues during development—not only the placenta but also the yolk sac and allantois—provided opportunities for the expression, retention, and eventual co-option of virus-derived sequences. The involvement of *SIRH3/RTL6*, *SIRH8/RTL5*, *SIRH10/RTL9*, *SIRH11/RTL4*, and *ERVPb1* in microglia and other tissue-resident macrophage lineages is consistent with this idea. Once established in the host genome, such virus-derived sequences could subsequently be recruited into a wide range of biological systems [36].

**Figure 7:**
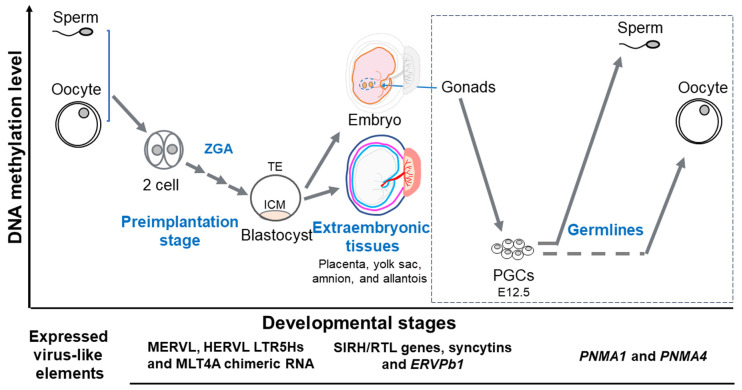
Low-DNA-methylation developmental environments in eutherian mammals: Genome-wide demethylation occurs during preimplantation development, including the zygotic genome activation (ZGA) stage, reaching its lowest level at the blastocyst stage. After implantation, de novo DNA methylation is established in the embryo proper, whereas extraembryonic tissues, including the placenta, yolk sac, and allantois, remain relatively hypomethylated. Genomic imprinting marks are completely erased in primordial germ cells (PGCs) by embryonic day 12.5 [80,81]. Male-specific imprinting marks are established before birth, whereas female-specific imprinting marks are progressively acquired during oocyte growth and are largely completed by ovulation. ICM: inner cell mass; TE: trophectoderm; orange: ICM; light blue, pink, and dark blue lines around embryo are amnion, visceral yolk sac, and parietal yolk sac, respectively. The thick red line connecting the embryos and the placenta represents the umbilical cord, which is derived from the allantois. The following papers were referenced when creating this figure [73,74,75].

The importance of DNA methylation in repressing retroviruses and retrotransposons is well-recognized. The loss of DNMT1, the major DNA methylation enzyme, results in embryonic lethality associated with an increase in ERV expression due to the demethylation of viral LTR promoters [69,70,71]. Additionally, B-cell transformation caused by EBV has been reported to lead to genome-wide DNA hypomethylation, activating the LTR promoters and enhancers of ERVs [72]. In eutherian development, other environments characterized by low DNA methylation include the germline and preimplantation developmental stages [73,74,75,76,77,78,79] (Figure 7). Following fertilization, extensive genome-wide demethylation occurs during preimplantation development, and DNA methylation levels reach their lowest levels at the blastocyst stage. After implantation, de novo methylation is established in the embryo proper, whereas extraembryonic tissues, including the placenta, yolk sac, and allantois, remain relatively hypomethylated. The germline in embryos is subject to specific DNA methylation regulation. Genome-wide DNA demethylation occurs alongside the erasure of genomic imprinting marks, a process that is essential for mammalian development [76,79,80,81]. Subsequently, DNA remethylation of imprinting control regions occurs at different developmental stages in the male and female germline lineages. Methylation patterns in male germ cells are established before birth. In contrast, in female germ cells, these patterns are gradually acquired during oocyte growth and are largely complete by ovulation [73,74,75]. These developmental windows collectively define the hypomethylated environments in which virus-derived sequences are most likely to be expressed, retained, and ultimately co-opted during mammalian evolution.

### 3.3. Emerging Perspectives on Mammalian Virus-Derived Genes

The PNMA gene family is another group of mammalian-specific metavirus-derived genes containing approximately 15 genes in humans and mice. They were originally identified as a family of genes expressed predominantly in the nervous system [82,83,84]. However, recently, *PNMA1* and *PNMA4* have been reported to be expressed in gonadal tissues and to play critical roles in the maintenance of germline cells [85]. These findings support the notion that PNMA1 and PNMA4 contribute to the maintenance of female reproductive capacity through their functions in germline cells. *PNMA1* and *PNMA4* are regulated by germ cell-specific factors, such as MYBL1, STRA8, and DAZL, during gametogenesis. These findings are consistent with the notion that virus-derived genes have been functionally integrated into germline-associated hypomethylated developmental environments (Figure 7). This finding raises the possibility that the evolutionary history of the PNMA gene family may parallel that of the SIRH/RTL family. For example, *PEG10* and *PEG11/RTL1*, which were initially recruited for essential functions in the placenta [7,8], were subsequently co-opted for important functions in the nervous system [19,20,21,22,23]. Likewise, because many members of both the PNMA family and the SIRH/RTL group are highly expressed in the gonads and placenta, it is conceivable that some PNMA genes were initially selected for functions in reproductive tissues and only later acquired additional roles in the nervous system. Conversely, the reproductive functions identified for *PNMA1* and *PNMA4* raise the possibility that additional members of the SIRH/RTL family may also contribute to reproductive processes. Among the PNMA family members, only a limited number have been linked to defined organismal phenotypes. *PNMA10* has been proposed as a candidate gene for X-linked intellectual disability [86], whereas *PNMA14* (*CCDC8*) is responsible for 3-M syndrome, a rare autosomal recessive growth disorder characterized by severe pre- and postnatal growth impedance, short stature, characteristic craniofacial features, and skeletal abnormalities [87,88]. Therefore, determining the tissues and biological functions that originally drove the evolutionary retention of these metavirus-derived genes will provide important insights into their origins and diversification. It is also possible that functional interactions within and between metavirus-derived and retrovirus-derived gene networks contributed to the emergence of novel biological systems during mammalian evolution.

Furthermore, retroviruses and retrovirus-derived LTR sequences, such as murine endogenous retrovirus-L (MERVL) and human endogenous retrovirus K (HERVK)-derived LTR5Hs, function as important promoters and enhancers during preimplantation development from the two-cell stage to the blastocyst stage [89,90]. Recently, chimeric RNAs containing transcripts from MLT2A1, one of the ERVs, function as important transcriptional activation factors in the major zygotic genome activation (ZGA) stage [91]. This suggests that hypomethylated developmental environments may also facilitate the functional co-option of virus-derived sequences (Figure 7).

Together, these observations suggest that hypomethylated developmental environments repeatedly provided opportunities for the expression, retention, and functional integration of virus-derived sequences during eutherian evolution.

Genome-wide analyses have suggested that the human genome contains tens of thousands of endogenous viral element-derived open reading frames (ORFs), including many retrovirus-derived sequences encoding proteins longer than 80 amino acids [92,93,94]. Additional virus-derived domesticated genes involved in innate immunity, similar to *ERVPb1*, are therefore likely to be identified in the future. Understanding the functions of these genes will be important not only for elucidating the evolution of mammalian-specific innate immune systems, but also for clarifying the molecular mechanisms underlying human neurological and psychiatric disorders. Future studies integrating evolutionary genomics, developmental epigenetics, and functional genetics will be essential for testing this expanded framework of placenta-driven evolution.

## Data Availability

No new data were created or analyzed in this study. Data sharing is not applicable to this article.

## Supplementary Materials

The following supporting information can be downloaded at: https://www.mdpi.com/article/10.3390/ijms27146322/s1.

## Abbreviations

The following abbreviations are used in this manuscript:

SIRH	sushi-ichi retrotransposon homologues
RTL	Retrotransposon Gag-like
LTR	long terminal repeat
ERVs	endogenous retroviruses
KI	knock-in
KO	knockout
LPS	lipopolysaccharide
dsRNA	double-stranded RNA
PAMPs	pathogen-associated molecular pattern
ASD	Autism spectrum disorder
NA	Noradrenaline
